# Supplementary material for: Recombinant AAV-CEA Tumor Vaccine in Combination with an Immune Adjuvant Breaks Tolerance and Provides Protective Immunity
Source: Mol Ther Oncolytics. 2018 Dec 13;12:41–8. doi: 10.1016/j.omto.2018.12.004 (PMC6329706; doi:10.1016/j.omto.2018.12.004)
Supplement: Document S1. Figure S1 [file mmc1.pdf]

OMTO, Volume *12*

Supplemental Information

Recombinant AAV-CEA Tumor Vaccine in Combination with an Immune Adjuvant Breaks Tolerance and Provides Protective Immunity

Jonathan A. Hensel, Vinayak Khattar, Reading Ashton, and Selvarangan Ponnazhagan

Supplemental Figure 1

Antibody Control PD-L1

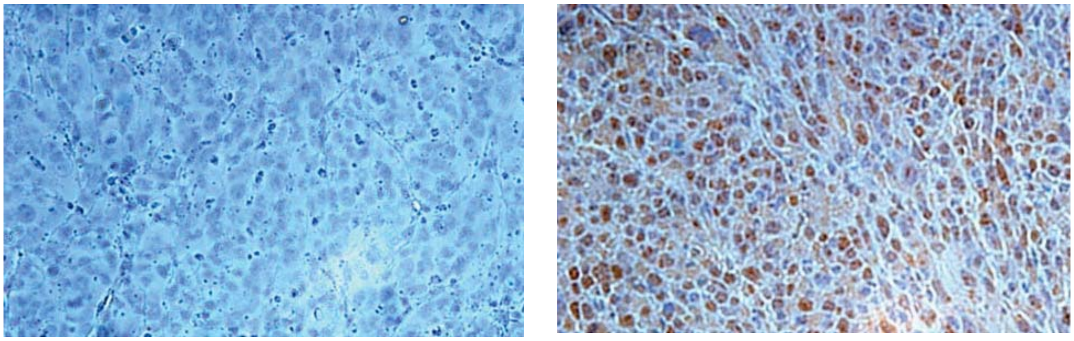

PD-L1 expression in MC38-CEA tumor. Representative immunohistochemistry analysis indicates high expression of PD-L1 in a mouse with resistant tumor growth, within the rAAV-CEA treatment group.
