# Supplementary material for: Recombinant AAV-CEA Tumor Vaccine in Combination with an Immune Adjuvant Breaks Tolerance and Provides Protective Immunity
Source: Mol Ther Oncolytics. 2018 Dec 13;12:41–8. doi: 10.1016/j.omto.2018.12.004 (PMC6329706; doi:10.1016/j.omto.2018.12.004)
Supplement: Document S2. Article plus Supplemental Information [file mmc2.pdf]

Molecular Therapy
Oncolytics
Original Article

Recombinant AAV-CEA Tumor Vaccine in Combination with an Immune Adjuvant Breaks Tolerance and Provides Protective Immunity

Jonathan A. Hensel,[1] Vinayak Khattar,[1] Reading Ashton,[1] and Selvarangan Ponnazhagan[1]

[1]Department of Pathology, The University of Alabama at Birmingham, Birmingham, AL 35294, USA

Carcinoembryonic antigen (CEA) is a human glycoprotein involved in cellular adhesion and expressed during human fetal development. Although expression of CEA largely ceases prior to birth, several human epithelial cancers, including colorectal, gastric, squamous esophageal, and breast carcinomas have been known to overexpress CEA, suggesting its potential as an immunotherapeutic target. Using a transgenic mouse model constitutively expressing human CEA in a spatiotemporal manner as a self-protein and a syngeneic mouse colon cancer cell line, MC38-CEA, overexpressing CEA, we tested the potential of a novel genetic immunotherapy approach against CEA-expressing tumors, using recombinant adeno-associated virus vector encoding CEA (rAAV-CEA) and appropriately timed immune adjuvant application. Results of the study demonstrated breaking of immune tolerance for CEA with this vaccine regimen and an anti-tumor response, resulting in tumor-free survival. Furthermore, tumor challenge of CEA-vaccinated mice with parental MC38 cells not expressing CEA did not result in protection from tumor development, confirming that the protection against tumor development is CEA specific. The study illustrates the feasibility of utilizing rAAV vectors in combination with an immunostimulatory adjuvant to break tolerance to weakly immunogenic self-antigens and for an anti-tumor response.

INTRODUCTION

Carcinoembryonic antigen (CEA) is a human glycoprotein involved in cellular adhesion and is expressed during human fetal organ development. Following birth, the expression of CEA is largely dampened, with only low levels in the plasma of healthy adults.[1] However, CEA is overexpressed in many human cancers, including gastric, colorectal, breast, ovarian, lung, and pancreatic cancers.[2] As it is a cellular adhesion molecule, its high expression in these cancers can promote increased intercellular adhesion, in turn supporting the metastatic process.[2] These characteristics make CEA a logical target for vaccine-based immunotherapeutics, attempting to break tolerance within CEA-expressing tumors.[3]

There is a multitude of potential viral vectors currently being tested for the development of cancer vaccines. Among these, the recombinant adeno-associated virus vector (rAAV) holds great promise and circumvents many of the shortcomings of other vectors such as adenoviral, lentiviral, and retroviral vectors regarding clinical safety and immunological clearance prior to sufficient transgene expression. Unlike retroviral vectors and the subset lentiviral vectors, rAAV rarely integrates into the host genome,[4,5] which greatly reduces the risk of insertional mutagenesis. Further, AAV is not associated with any known disease and, in fact AAV has been reported to have anti-oncogenic properties against human papillomavirus (HPV)-induced carcinogenesis.[6–8] Preclinical studies using AAV vectors for cancer therapy have begun to show promise.[9] The salient features of AAV vectors, including low vector-based immunogenicity and efficient transgene expression, are now being utilized in several cancer clinical trials.[9] Vaccines targeting tumor-associated antigens often fail to elicit clinical efficacy due to insufficient immune response.[10] A low dose of tumor antigen has been shown to favor a Th2 response, whereas a higher dose has been shown to favor Th1 response, required for eliciting a strong T cell immunity against tumors.[11] In this context, rAAV remains an ideal vector to achieve high antigen expression. Additional immunological stimulation has been demonstrated as a requisite to provide an immune response sufficient to break tolerance against "self" antigens.[2,3,12] For a strong cellular response, it is necessary to stimulate cross-presentation of rAAV-encoded tumor-associated antigens (TAAs) to activate cytotoxic T lymphocyte (CTL) response against tumors. Granulocyte-macrophage colony-stimulating factor (GM-CSF) is a potent chemoattractant of antigen presenting cells (APC) in this regard.[13]

Using an rAAV expressing CEA antigen and a plasmid adjuvant encoding GM-CSF, we, for the first time to our knowledge, demonstrate the efficacy of an rAAV-CEA vaccine against a syngeneic, CEA-expressing, gastrointestinal cell line tumor in a CEA transgenic mouse model that constitutively expresses CEA in a spatiotemporal manner. The results of the study demonstrated the breaking of immune tolerance to CEA in this model and verified that an rAAV vaccine can provide antigen-specific anti-tumor response.

Received 11 November 2018; accepted 7 December 2018;
https://doi.org/10.1016/j.omto.2018.12.004.

Correspondence: Selvarangan Ponnazhagan, Ph.D., Department of Pathology, The University of Alabama at Birmingham, SHEL 814, 1825 University Blvd., Birmingham, AL 35294, USA.
E-mail: sponnazh@uabmc.edu

This is an open access article under the CC BY license (http://creativecommons.org/licenses/by/4.0/).

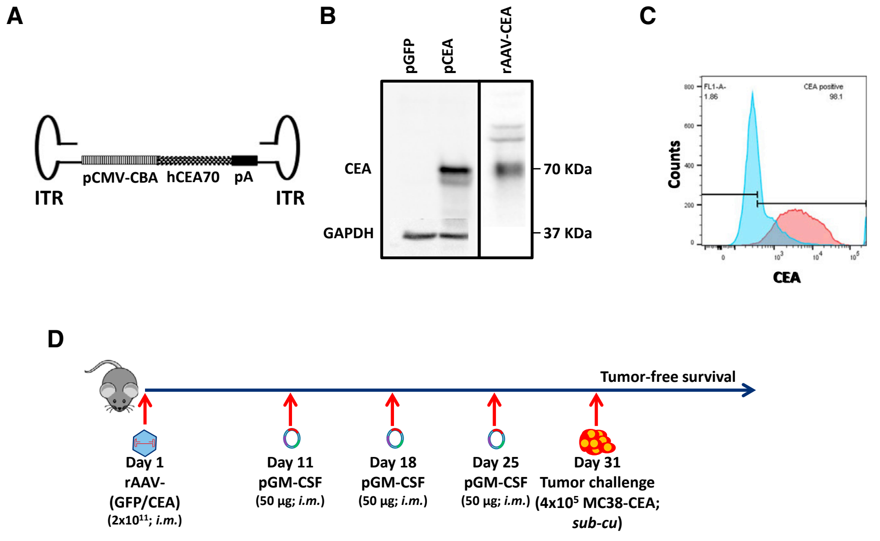

Figure 1. Characterization of Recombinant-AAV Expressing Human CEA, the C57BL/6 Syngeneic Cell Line MC38-CEA, and the Experimental Outline

(A) The rAAV-CEA construct was developed by sub-cloning the human CEA70 open reading frame under the control of cytomegalovirus (CMV)-chicken beta-actin promoter and poly(A) sequence within AAV1 inverted terminal repeats. (B) Western blotting for CEA expression in HEK293 cells, either transfected with pAAV-CEA or transduced with rAAV-CEA. Western blot images presented are a composite of results from two different blots, in each box. In the plasmid transfection experiment (left), the same blot was used for staining with CEA and GAPDH antibodies in succession. (C) Flow cytometry of CEA expression in C57BL/6 syngeneic colon cancer cell lines: MC38 parental cells (blue histogram) and MC38-CEA (red histogram). (D) Overall experimental schema: on day 1, mice were either unvaccinated or intramuscularly vaccinated once with either rAAV-GFP or rAAV-CEA (2×10^{11} virus particles per mouse). Following vector vaccinations, on days 11, 18 and 25, mice were given 50 μg GM-CSF at the rAAV vaccination sites. On day 31, mice were challenged with 4×10^5 syngeneic MC38-CEA cells.

RESULTS

Characterization of rAAV Expressing Human CEA70 and the C57BL/6 Syngeneic Cell Line MC38-CEA

To test our hypothesis that a robust transgene expression capability of AAV vectors can be exploited for breaking immune tolerance to a "self" antigen and to function as an efficacious cancer vaccine, we created a recombinant AAV (rAAV) by first sub-cloning human CEA (hCEA) into the open reading frame of pAAV-MCS plasmid under the control of a human cytomegalovirus (CMV)-chicken beta-actin hybrid promoter (Figure 1A). This rAAV plasmid was then used for the production of virions by co-transfection with the transgene-packaged plasmid along with helper plasmids encoding adenovirus and AAV genes required for the rescue, replication, and packaging of rAAV-CEA genome in HEK293 cells, followed by harvest and purification.[14] Western blot analysis using lysates from HEK293 cells transfected or transduced with the CEA plasmid and rAAV-CEA virus, respectively, confirmed the expression of CEA (Figure 1B). Further, the syngeneic MC38-CEA gastrointestinal cell line, constitutively expressing human CEA, was tested by flow cytometry for CEA expression. Overlaid histograms of CEA expression in parental MC38 and MC38-CEA demonstrated homogeneous expression of CEA within MC38-CEA cells (Figure 1C). Following the development of rAAV-CEA and control rAAV-GFP, a prophylactic tumor model was developed to test the efficacy of rAAV-CEA vaccination plus GM-CSF adjuvant as outlined (Figure 1D).

Immunohistochemistry for CEA and Immune Cell Infiltrates at the Site of Vaccination

Based on our hypothesis that robust CEA antigen expression and an optimal immune adjuvant stimulus are needed for breaking immune tolerance, the rAAV-CEA vaccine was injected intramuscularly into CEA-Tg mice. A plasmid expressing GM-CSF was administered to the site on days 10, 17, and 21 post-rAAV-CEA applications and compared to control groups. The influx of immune cells following adjuvant administrations was verified from respective cohorts of mice. Muscle tissues from controls and CEA-vaccinated mice at the site of vaccination were fixed and stained by immunohistochemistry to examine immune cell infiltration. Staining of sections with CEA antibody demonstrated the presence of CEA expression in rAAV-CEA + GM-CSF, whereas rAAV-GFP + GM-CSF-vaccinated mouse tissue did not express CEA (Figure 2A). This demonstrated the efficacy of the rAAV-CEA vaccine at producing CEA *in vivo*. Staining muscle tissues for CD45, a hematopoietic cell marker, showed that an influx of immune cells was largely confined to mice receiving the combination treatment of rAAV-CEA vaccine + GM-CSF, as compared to mice vaccinated with rAAV-CEA alone (Figure 2B). Characterization of the immune cell infiltrates identified infiltration of macrophages using F4/80 antibody in the rAAV-CEA + GM-CSF cohort, compared to rAAV-CEA alone (Figure 2B). This confirmed that rAAV vector alone was not sufficient to elicit prolonged inflammation at the vector injection site at a time when optimal transgene expression is achieved and that adjuvant GM-CSF injections are required to achieve optimal immune infiltration for cross-presentation. These experiments demonstrated that rAAV-CEA + GM-CSF is effective at both CEA expression and chemoattraction of immune cells, including macrophages, capable of CEA cross-presentation to T cells.

rAAV-CEA Plus GM-CSF Adjuvant Breaks Tolerance to CEA "Self" Antigen

As a measure of analyzing the breaking of immune tolerance to CEA on a humoral level, we measured CEA antibodies by

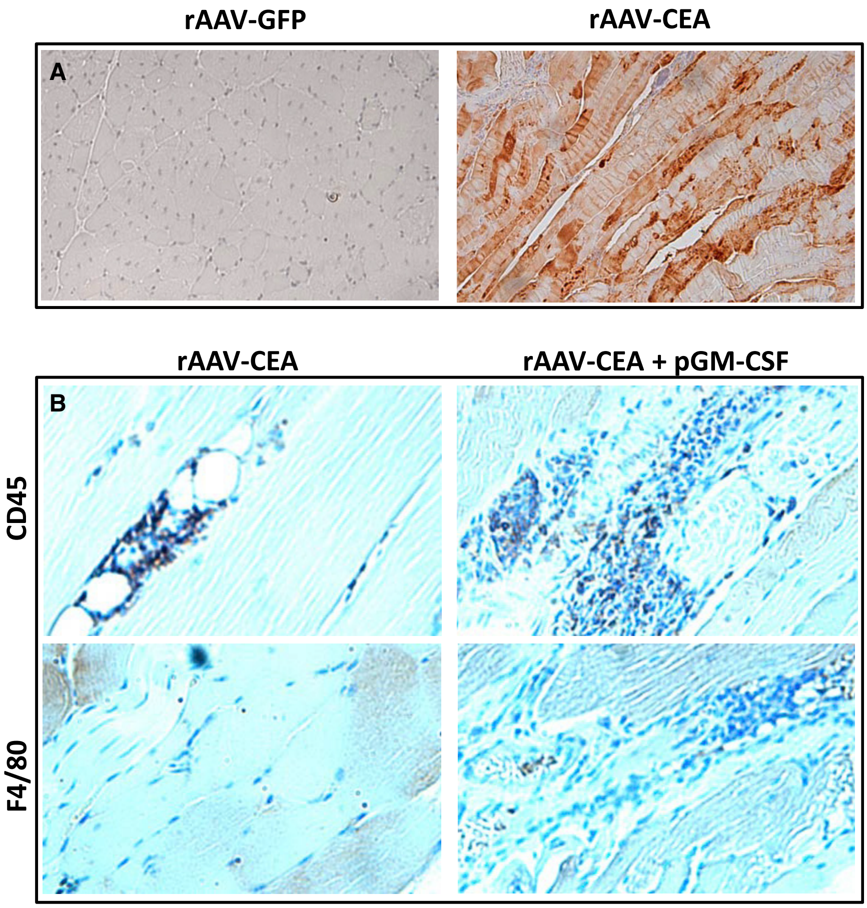

Figure 2. Immunohistochemistry for CEA Expression and Immune Infiltrates at the Site of Vaccination

(A) Immunohistochemistry for CEA was performed in muscle tissue of rAAV-GFP or -CEA-vaccinated mice. (B) Muscle tissues from mice that received rAAV-CEA alone or rAAV-CEA + pGM-CSF immune adjuvant were fixed and stained by immunohistochemistry with CD45 antibody for overall immune infiltrates and with F4/80 antibody for macrophages in particular.

ELISA from plasma samples collected from animals, post-rAAV-CEA vaccination, plus GM-CSF adjuvant, but prior to tumor challenge. Results of the ELISA demonstrated that the vaccination of animals with rAAV-CEA + GM-CSF led to a significant increase in anti-CEA antibody production (Figure 3; $n = 3$, $p < 0.01$).

Tumor-free Survival in Response to rAAV-CEA Vaccination

Following completion of the vaccine protocol and tumor challenge as outlined earlier (Figure 1D), mice were monitored for tumor-free survival. Results of this study indicated that, in the rAAV-CEA + GM-CSF group, there was a reduction in tumor development, providing significant long-term tumor-free survival even after a time point that was nearly double the time during which all mice in the naive and rAAV-GFP cohorts had developed tumors (Figure 4; $n = 5$, $p < 0.01$)

Vaccine Efficacy of rAAV-CEA Plus GM-CSF Regimen Is Antigen-Specific

After establishing that vaccination provided significant breaking of humoral tolerance and offered tumor-free survival against CEA-expressing tumors, we next tested whether the anti-tumor effect of rAAV-CEA vaccination was antigen-specific, using the parental version of MC38-CEA cells that lack CEA expression (MC38). Flow cytometry analysis, comparing CEA expression in MC38 cells and MC38-CEA cells, confirmed the lack of CEA expression in MC38 cells (Figure 5A). Following the rAAV-CEA vaccination plus GM-CSF adjuvant strategy, mice were tumor challenged with 4×10^5 MC38 cells and monitored for tumor development. As GM-CSF was seen to provide delayed tumor development in previous experiments, a GM-CSF-only cohort and a naive cohort with only tumor challenge and no rAAV-CEA served as controls. All of the rAAV-CEA-vaccinated and GM-CSF adjuvant injected groups developed tumors at early time points with no significant delay when compared to GM-CSF vaccinated and the naive groups. These results demonstrate that the efficacy of rAAV-CEA vaccination plus GM-CSF adjuvant, observed in previous experiments, was CEA specific (Figure 5B; $n \geq 3$).

DISCUSSION

The recent promise of AAV vectors in human gene therapy has generated renewed interest for examining its use in the context of cancer immunotherapy. Although robust and durable expression of rAAV-encoded transgenic proteins is an attractive property for mounting an effective anti-tumor cellular response, the low vector immunity and kinetics of rAAV-transgene expression precludes utilization of this non-pathogenic vector system for cancer vaccine development. To overcome this limitation, we hypothesized that providing a secondary immune adjuvant signal at the vector-transduced tissue site when optimal CEA expression was achieved would overcome this limitation. Testing of this protocol in CEA Tg mice, in fact, indicated the breaking of immune tolerance of CEA by this combination, a key requisite for targeting many human cancers by immunotherapy, characterized by overexpression of tumor antigens.

The importance of establishing a cellular immune response to tumor antigens is vital in forming efficacious anti-tumor responses using peptide-based vaccines.[15,16] Comparison of tumor inhibition

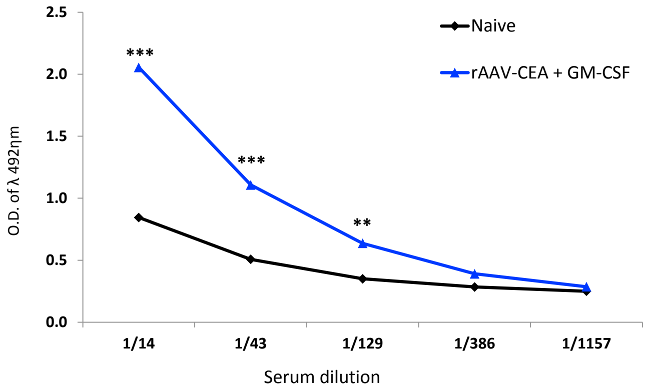

Figure 3. ELISA for CEA as a Measure of Breaking Tolerance to a Self Antigen

CEA ELISA was performed in replicates using serum samples from naive mice and mice that received rAAV-CEA + pGM-CSF adjuvant (n = 3). $^{**}p < 0.01$; $^{***}p < 0.001$.

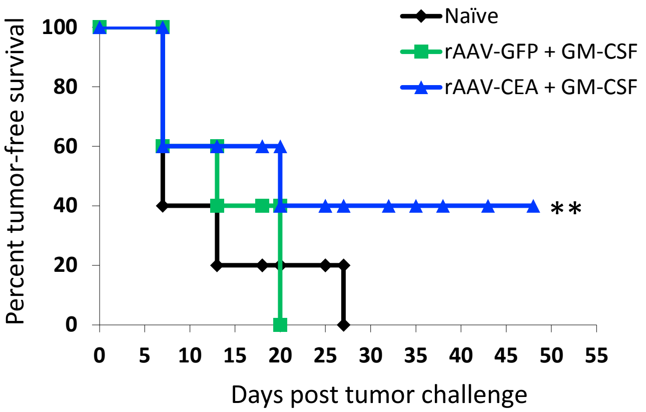

Figure 4. Tumor-free Survival in Response to rAAV-CEA Vaccine Plus pGM-CSF

Following rAAV-CEA vaccination and application of pGM-CSF at the site of vector administration, when optimal CEA expression was achieved, mice were challenged with CEA-expressing, syngeneic MC38-CEA cells (4×10^5) in the flank region. Tumor-free survival of naive, rAAV-GFP + pGM-CSF, or rAAV-CEA + pGM-CSF mice was recorded well beyond the time when mice in control groups had developed tumors (n = 5). $^{**}p < 0.01$.

following treatment with anthracyclines among T cell-deficient mice versus mice with antibody-depleted B cells has shown poor results among animals lacking T cells, whereas efficacy was still apparent within the B cell-depleted mice, implying a crucial role for T cells in anti-tumor therapies.[17] While CTLs serve a requisite role in the anti-tumor activity in genetic-based immunotherapy, studies have also confirmed the need to augment CTL activation using antigen-presenting cells (APCs). Their uptake, processing, and cross-presentation of antigens to tumor-specific CTLs make them indispensable contributors to an effectual vaccine-based anti-tumor therapy.[12,15,18–21] GM-CSF adjuvant has proven effective as an adjuvant for increasing APC intratumoral infiltration, thus permitting antigen uptake and cross-presentation to CTLs.[18,22] In addition to CTL cellular responses against tumor cells and augmented APC cross-presentation, a third component for a successful anti-tumor peptide-based vaccination is the initiation of a Th2 humoral response.[22–27] Though CTL responders are now seen as critical components of vaccine-based therapies, the humoral arm should not be ignored.

Cancer has proven a difficult disease to fully and permanently eradicate in patients, as has been observed repeatedly with conventional strategies relying on surgery, chemo, and radiation therapies. This has much to do with its adaptability and/or the survivability of subsets of cancer cells within heterogeneous tumors possessing distinct genetic signatures that permit therapy circumvention and eventual tumor outgrowth.[28–31] In 2016, the "Cancer Moonshot" was launched, with the aim to find cures for cancer relying heavily on the promise of immunotherapy.[32] As immunotherapies evolve, it is vital that we understand the need to include all the arms of the immune system, as they have multi-directional influence on vaccine efficacy. In this context, the aim of the present study was to improve APC infiltration into the vaccination site for efficient tumor antigen uptake, processing, and presentation for a strong anti-tumor cellular response while also breaking tolerance to a "self" antigen, which is the case in many passive immunotherapy strategies.

The present study addresses the important points of improved APC infiltration into the site of vaccination and breaking of immune tolerance. Results of immunohistochemistry demonstrated not only effective delivery of CEA through rAAV but also that GM-CSF adjuvant application improved immune infiltrates into the vaccine site. ELISA for CEA showed rAAV-CEA results in breaking of humoral tolerance with the production of anti-CEA antibody that corroborated with a significant anti-tumor activity in mice, which was also confirmed to be CEA specific. These points denote that use of rAAV vectors can provide an antigen-specific anti-tumor response.

Despite significant protection against tumor growth following rAAV-CEA immunotherapy, some mice developed tumors in this group. A possible explanation for tumor development within this cohort is a noted robust intratumoral expression of programmed death-ligand 1 (PD-L1), (Figure S1). This prompts future studies combining checkpoint inhibitors targeting PD1 and/or PD-L1 to further improve anti-tumor response. Recent studies have reported that the Th1 cytokine, interferon (IFN)-gamma, can lead to upregulation of PD-L1 by tumors.[33,34] Counter intuitively, though previously viewed as being an anti-tumor cytokine, IFN-gamma appears to regulate a pro-tumoral mechanism, increasing PD-L1 expression through the JAK-STAT pathway.[35] Furthermore, in gastric cancer, this increased PD-L1 expression correlates with the presence $CD8^+$ T cells in the tumor stroma.[35] Therefore, combining rAAV-CEA vaccination with an anti-PD-L1 adjuvant to address this point should further improve vaccine efficacy.

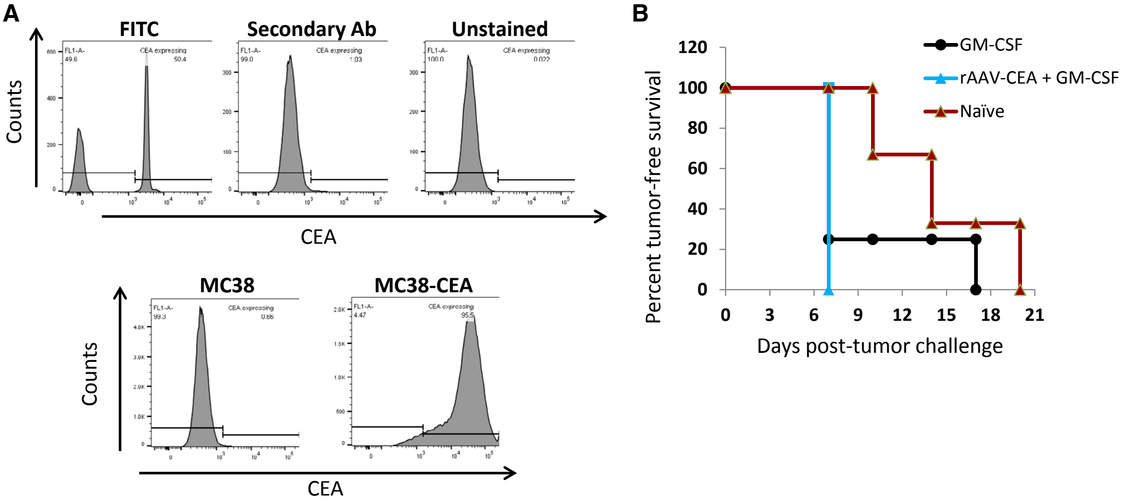

Figure 5. Expression of CEA by MC38 Parental Cells and CEA-Expressing MC38-CEA Cells and Tumor Challenge Study with MC38 Parental Cells following rAAV-CEA + pGM-CSF Adjuvant Applications

(A) CEA expression on MC38 parental cells was compared to that expressed by MC38-CEA cells to confirm lack of CEA expression by parental cells. (B) Cohorts of naive mice, mice that received pGM-CSF adjuvant injections only, or mice that were vaccinated with rAAV-CEA plus pGM-CSF adjuvant injections were tumor challenged with MC38 parental cells (4×10^5) and assessed for tumor development, starting 1 week post-tumor challenge ($n \geq 3$).

The vaccine approach of the study has clear clinical application. It is estimated that up to 30% of all colorectal cancers are an inheritable disease.[36,37] CEA is so commonly expressed by colorectal cancers (89%), it is the favored biomarker for this type of cancer.[2,38] Additionally, among sporadic colorectal cancers, approximately 15% have pervasive microsatellite instabilities (MSIs) that promote transformation.[39] Therefore, targeting patients having familial mutations or those with MSIs that predisposes them to a higher risk for colorectal cancer might lead to benefit from prophylactic vaccination using AAV-based vectors expressing CEA. As CEA has been a long sought-after vaccine target in multiple cancer types, our findings are important, as they, for the first time, demonstrate the feasibility of anti-tumor responses in CEA-expressing tumors using AAV, a vector not associated with any pathology and that possesses minimal risk for insertional mutations, overcoming obstacles that have been viewed as major safety concerns with other viral vectors.

MATERIALS AND METHODS

Mice

Six- to 8-week-old female and male pathogen-free CEA transgenic mice in a C57BL/6 background (C57BL/6J-TgN(CEAGe)18FJP) previously acquired from Dr. James F. Primus at Vanderbilt University Medical Center, were bred and housed in accordance with established guidelines and protocols approved by The University of Alabama at Birmingham Institutional Animal Care and Use Committee (UAB IACUC).

Cells and Reagents

HEK293 cells were purchased from ATCC (Manassas, VA, USA) and cultured in DMEM from GIBCO-Life Technologies (Grand Island, NY, USA) with 1% penicillin-streptomycin (Pen Strep; GIBCO) supplemented with 10% heat-inactivated fetal bovine serum (FBS) from Omega Scientific (Tarzana, CA, USA) in a 5% CO_2 37°C humidified incubator. 0.25% Trypsin-EDTA (GIBCO) was used to detach cells from culture plates. The murine adenocarcinoma cell line, MC38-CEA, was obtained from Dr. Jeffrey Schlom from the National Cancer Institute (Bethesda, MD, USA) and cultured in DMEM supplemented with 1% non-essential amino acids, 1% L-glutamine, 1% sodium pyruvate, 1% Pen Strep, 400 μg/mL Geneticin (all from GIBCO), and 10% heat-inactivated FBS (Omega Scientific). Human CEA protein was purchased from Fitzgerald Industries International (Concord, MA, USA). Mouse monoclonal antibody, (COL-1 clone) to human CEA was purchased from Invitrogen (Rockford, IL, USA). Mouse immunoglobulin G (IgG) control (catalog #sc-2027) was purchased from Santa Cruz Biotechnology (Dallas, TX, USA). Secondary anti-mouse antibody, labeled with horseradish peroxidase (HRP), was purchased from Invitrogen (catalog #31430) or GE Healthcare (catalog #NA931V; Marlborough, MA, USA). FcR block (catalog #130-092-575) was purchased from Miltenyi Biotec (San Diego, CA, USA). Unlabeled anti-CEA, COL-1 (COL-1 clone), and biotin-labeled anti-CEA (COL-1 clone) were purchased from Invitrogen. Biotin-labeled IgG control was purchased from BioLegend (MOPC-173 clone; San Diego, CA, USA). Fluorescein isothiocyanate (FITC)-labeled streptavidin was purchased from BioLegend (catalog #405201). A secondary, FITC-labeled anti-mouse IgG antibody (catalog #A21202) was purchased from Invitrogen. APC-Cy7-labeled CD3e antibody (145-2C11 clone) was purchased from BD Pharmingen (San Jose, CA, USA). FITC-labeled CD8 (536.7 clone), APC-labeled CD44 (IM7.8.1 clone), and VioBlue-labeled CD62L (MEL14-H2.100 clone) were all purchased from Miltenyi Biotec. Unlabeled CD45 (30-F11 clone), CD11b (M1/70.15 clone), F4/80 (BM8 clone), and CD19 (60MP31

clone) antibodies were purchased from Invitrogen. AAV serotype 1 (AAV1) was used for the vaccine.

Western Blot Analysis

Cells were harvested for protein isolation using a lysis buffer containing protease (Roche Diagnostics, Indianapolis, IN, USA) and phosphatase (Thermo Scientific, Rockford, IL, USA) inhibitors. Following denaturation, the samples were separated on a 10% polyacrylamide gel and transferred overnight to a polyvinylidene fluoride (PVDF) membrane (Bio-Rad, Hercules, CA, USA), followed by blocking using 5% non-fat milk and incubation with primary antibodies. Following overnight incubation with primary antibody at 4°C and subsequent washes (3 × 10 min) with 1× Tris-buffered saline with Tween 20 (TBST), an appropriate HRP-conjugated secondary antibody was applied for 1 h at room temperature (GE Healthcare) and then washed with TBST (3 × 10 min).[40,41] Blots were then incubated with chemiluminescence reagent (GE Healthcare) according to the manufacturer's directions and developed in a Syngene PXi chemiluminescence developer (Frederick, MD, USA).

Immunohistochemistry

Immunohistochemistry was performed on 5-μm sections of paraffin-embedded tumor tissues. After rehydration, slides were incubated with citrate buffer for 20 min in a steamer for antigen retrieval and endogenous peroxidase quenched by incubation with 3% H_2O_2 for 20 min at room temperature. Primary antibody incubation was performed overnight at 4°C. Secondary antibodies used were linked to HRP and incubated with samples for 1 h at room temperature. Visualization of the bound complex was performed using diaminobenzidine tetrahydrochloride. The slides were counterstained minimally with hematoxylin.

ELISA for CEA

ELISA was performed for serum CEA antibody detection. 96-well ELISA flat-bottom plates (Costar 3590; Corning, Acton, MA, USA) were coated with 100 μL human CEA protein (Fitzgerald Industries) at a concentration of 1 mg/mL in PBS and incubated overnight at 4°C. Ovalbumin-coated wells (negative control) were treated in the same manner. Following overnight incubation, plates were emptied and gently slapped onto absorbent towels to remove the residual solution, and then were blocked with 1% borate saline (BS)-BSA solution for 1.5 h, followed by washing 3× using a PBS-0.05% Tween 20 solution. 3-fold serial dilutions (from 1/50 to 1/109,350) of mouse serum using 1% BS-BSA were then applied to wells in triplicate overnight at 4°C. COL-1 mouse monoclonal antibody to human CEA (Invitrogen) in place of serum was used as a positive control. Plates were then emptied or gently slapped onto absorbent towels and washed 3× using PBS-0.05% Tween 20 solution, and then incubated with HRP-conjugated goat anti-mouse IgG from Invitrogen (catalog #31430) diluted 1:750 in BS-BSA for 6 h at room temperature in the dark. After washing 3×, 125 μL SIGMAFAST solution from Sigma-Aldrich (Burlington, MA, USA) was applied to each well and incubated at room temperature for 30 min in the dark. Absorbance was then immediately measured at 492λ on a BioTek Instruments plate reader using Gen5 software (Winooski, VT, USA).

Tumor Cell Implantation and Evaluation of Tumor-free Survival in Response to rAAV-CEA Vaccination

Following intra-muscular vaccination with 2×10^{11} vector particles of either rAAV-GFP or rAAV-CEA and 3 weekly GM-CSF (50 μg per mouse per application) adjuvant injections at the vaccination site, mice were subcutaneously tumor challenged with 4×10^5 MC38-CEA cells and measured twice weekly for syngeneic tumor development, starting 1 week post-implantation.

Parental MC38 Tumor Challenge

In studies to establish CEA specificity of the genetic vaccine, the parental MC38 colon cancer cells that are devoid of CEA expression were implanted in mice (4×10^5 cells per mouse), after administering a rAAV-CEA plus adjuvant GM-CSF vaccination protocol in CEA transgenic mice, and were monitored for tumor development. Experimental controls were tumor-challenged mice that were administered either GM-CSF only or naive mice.

Flow Cytometry Analysis and FACS

MC38-CEA and parental MC38 cells were detached from culture plates by first removing culture media, followed by a gentle wash with PBS to remove residual culture media before incubation with 0.25% Trypsin-EDTA (GIBCO), and then were washed using PBS containing FBS to neutralize trypsin and pelletized. For all experiments, cells were re-suspended in fluorescence-activated cell sorter (FACS) buffer (PBS + 3% FBS). Cells were divided into individual tubes with 100 μL FACS buffer and stained with appropriate antibodies.[42,43] Flow cytometry data was acquired on a BD LSR II instrument (BD Biosciences, Franklin Lakes, NJ, USA) and analyzed using FlowJo software (FlowJo, Ashland, OR, USA), while sorted cells were isolated using FACS on a BD FACSAria instrument (BD Biosciences).

Statistical Analysis

Results consisting of three groups were analyzed using single-factor ANOVA with Tukey test. Analysis of results containing two groups was performed using Student's t test. Values of $p \leq 0.05$ were considered statistically significant.

SUPPLEMENTAL INFORMATION

Supplemental Information includes one figure and can be found with this article online at https://doi.org/10.1016/j.omto.2018.12.004.

AUTHOR CONTRIBUTIONS

J.A.H. and S.P. designed study strategy; J.A.H., V.K., and R.A. performed experiments; J.A.H. performed analysis; J.A.H. and S.P. prepared the manuscript.

CONFLICT OF INTERESTS

The authors declare no competing interests.

ACKNOWLEDGMENTS

Financial support from NIH research grants CA184770 and AR060948 and Department of Defense grant PR141945 is greatly acknowledged. We also acknowledge the UAB Tissue Procurement Facility for providing cancer-associated fibroblasts.

REFERENCES

1. Adinolfi, M. (1998). Encyclopedia of Immunology, Second Edition (Elsevier), pp. 798–802.
2. Marshall, J. (2003). Carcinoembryonic antigen-based vaccines. Semin. Oncol. *30* (3, Suppl. 8), 30–36.
3. Berinstein, N.L. (2002). Carcinoembryonic antigen as a target for therapeutic anticancer vaccines: a review. J. Clin. Oncol. *20*, 2197–2207.
4. Nayerossadat, N., Maedeh, T., and Ali, P.A. (2012). Viral and nonviral delivery systems for gene delivery. Adv. Biomed. Res. *1*, 27.
5. Kotin, R.M., Linden, R.M., and Berns, K.I. (1992). Characterization of a preferred site on human chromosome 19q for integration of adeno-associated virus DNA by non-homologous recombination. EMBO J. *11*, 5071–5078.
6. Naso, M.F., Tomkowicz, B., Perry, W.L., 3rd, and Strohl, W.R. (2017). Adeno-associated virus (AAV) as a vector for gene therapy. BioDrugs *31*, 317–334.
7. Stilwell, J.L., and Samulski, R.J. (2003). Adeno-associated virus vectors for therapeutic gene transfer. Biotechniques *34*, 148–154.
8. Hermonat, P.L. (1994). Adeno-associated virus inhibits human papillomavirus type 16: a viral interaction implicated in cervical cancer. Cancer Res. *54*, 2278–2281.
9. Santiago-Ortiz, J.L., and Schaffer, D.V. (2016). Adeno-associated virus (AAV) vectors in cancer gene therapy. J. Control. Release *240*, 287–301.
10. Bodey, B., Bodey, B., Jr., Siegel, S.E., and Kaiser, H.E. (2000). Failure of cancer vaccines: the significant limitations of this approach to immunotherapy. Anticancer Res. *20*, 2665–2676.
11. Boonstra, A., Asselin-Paturel, C., Gilliet, M., Crain, C., Trinchieri, G., Liu, Y.J., and O'Garra, A. (2003). Flexibility of mouse classical and plasmacytoid-derived dendritic cells in directing T helper type 1 and 2 cell development: dependency on antigen dose and differential toll-like receptor ligation. J. Exp. Med. *197*, 101–109.
12. Ponnazhagan, S., Mahendra, G., Lima, J., Aldrich, W.A., Jenkins, C.B., Ren, C., Kumar, S., Kallman, L., Strong, T.V., Shaw, D.R., and Triozzi, P.L. (2004). Augmentation of antitumor activity of a recombinant adeno-associated virus carcinoembryonic antigen vaccine with plasmid adjuvant. Hum. Gene Ther. *15*, 856–864.
13. Zhu, X., Huang, X., and Zhang, W. (2000). [The promoting effects of mGM-CSF gene modified tumor vaccine immunization on peritoneal antigen presenting cells in mice]. Zhonghua Xue Ye Xue Za Zhi *21*, 591–594.
14. Ai, J., Ibraheim, R., Tai, P.W.L., and Gao, G. (2017). A scalable and accurate method for quantifying vector genomes of recombinant adeno-associated viruses in crude lysate. Hum. Gene Ther. Methods *28*, 139–147.
15. Jackson, S.R., Yuan, J., and Teague, R.M. (2014). Targeting CD8+ T-cell tolerance for cancer immunotherapy. Immunotherapy *6*, 833–852.
16. Maher, J., and Davies, E.T. (2004). Targeting cytotoxic T lymphocytes for cancer immunotherapy. Br. J. Cancer *91*, 817–821.
17. Hannani, D., Locher, C., Yamazaki, T., Colin-Minard, V., Vetizou, M., Aymeric, L., Viaud, S., Sanchez, D., Smyth, M.J., Bruhns, P., et al. (2014). Contribution of humoral immune responses to the antitumor effects mediated by anthracyclines. Cell Death Differ. *21*, 50–58.
18. Aldrich, W.A., Ren, C., White, A.F., Zhou, S.Z., Kumar, S., Jenkins, C.B., Shaw, D.R., Strong, T.V., Triozzi, P.L., and Ponnazhagan, S. (2006). Enhanced transduction of mouse bone marrow-derived dendritic cells by repetitive infection with self-complementary adeno-associated virus 6 combined with immunostimulatory ligands. Gene Ther. *13*, 29–39.
19. Morisada, M., Moore, E.C., Hodge, R., Friedman, J., Cash, H.A., Hodge, J.W., Mitchell, J.B., and Allen, C.T. (2017). Dose-dependent enhancement of T-lymphocyte priming and CTL lysis following ionizing radiation in an engineered model of oral cancer. Oral Oncol. *71*, 87–94.
20. Villadangos, J.A. (2016). Antigen-specific impairment of adoptive T-cell therapy against cancer: players, mechanisms, solutions and a hypothesis. Immunol. Rev. *272*, 169–182.
21. Zachova, K., Krupka, M., and Raska, M. (2016). Antigen cross-presentation and heat shock protein-based vaccines. Arch. Immunol. Ther. Exp. (Warsz.) *64*, 1–18.
22. Dela Cruz, J.S., Lau, S.Y., Ramirez, E.M., De Giovanni, C., Forni, G., Morrison, S.L., and Penichet, M.L. (2003). Protein vaccination with the HER2/neu extracellular domain plus anti-HER2/neu antibody-cytokine fusion proteins induces a protective anti-HER2/neu immune response in mice. Vaccine *21*, 1317–1326.
23. Castro-Díaz, N., Salaun, B., Perret, R., Sierro, S., Romero, J.F., Fernández, J.A., Rubio-Moraga, A., and Romero, P. (2012). Saponins from the Spanish saffron *Crocus sativus* are efficient adjuvants for protein-based vaccines. Vaccine *30*, 388–397.
24. Ioannides, C.G., Platsoucas, C.D., O'Brian, C.A., Patenia, R., Bowen, J.M., Wharton, J.T., and Freedman, R.S. (1989). Viral oncolysates in cancer treatment: immunological mechanisms of action (review). Anticancer Res. *9*, 535–544.
25. Neeson, P., Pan, Z.K., and Paterson, Y. (2008). Listeriolysin O is an improved protein carrier for lymphoma immunoglobulin idiotype and provides systemic protection against 38C13 lymphoma. Cancer Immunol. Immunother. *57*, 493–505.
26. Reilly, R.T., Emens, L.A., and Jaffee, E.M. (2001). Humoral and cellular immune responses: independent forces or collaborators in the fight against cancer? Curr. Opin. Investig. Drugs *2*, 133–135.
27. Jäger, D., Jäger, E., and Knuth, A. (2001). Immune responses to tumour antigens: implications for antigen specific immunotherapy of cancer. J. Clin. Pathol. *54*, 669–674.
28. Apicella, M., Corso, S., and Giordano, S. (2017). Targeted therapies for gastric cancer: failures and hopes from clinical trials. Oncotarget *8*, 57654–57669.
29. Chen, R., Smith-Cohn, M., Cohen, A.L., and Colman, H. (2017). Glioma subclassifications and their clinical significance. Neurotherapeutics *14*, 284–297.
30. Kerbel, R.S., and Shaked, Y. (2017). The potential clinical promise of 'multimodality' metronomic chemotherapy revealed by preclinical studies of metastatic disease. Cancer Lett. *400*, 293–304.
31. Kalkan, R. (2016). The importance of mutational drivers in GBM. Crit. Rev. Eukaryot. Gene Expr. *26*, 19–26.
32. Barlas, S. (2016). The White House launches a cancer moonshot: despite funding questions, the progress appears promising. P&T *41*, 290–295.
33. Mandai, M., Hamanishi, J., Abiko, K., Matsumura, N., Baba, T., and Konishi, I. (2016). Dual faces of IFNγ in cancer progression: a role of PD-L1 induction in the determination of pro- and antitumor immunity. Clin. Cancer Res. *22*, 2329–2334.
34. Zhang, X., Zeng, Y., Qu, Q., Zhu, J., Liu, Z., Ning, W., Zeng, H., Zhang, N., Du, W., Chen, C., and Huang, J.A. (2017). PD-L1 induced by IFN-γ from tumor-associated macrophages via the JAK/STAT3 and PI3K/AKT signaling pathways promoted progression of lung cancer. Int. J. Clin. Oncol. *22*, 1026–1033.
35. Mimura, K., Teh, J.L., Okayama, H., Shiraishi, K., Kua, L.F., Koh, V., Smoot, D.T., Ashktorab, H., Oike, T., Suzuki, Y., et al. (2018). PD-L1 expression is mainly regulated by interferon gamma associated with JAK-STAT pathway in gastric cancer. Cancer Sci. *109*, 43–53.
36. Lichtenstein, P., Holm, N.V., Verkasalo, P.K., Iliadou, A., Kaprio, J., Koskenvuo, M., Pukkala, E., Skytthe, A., and Hemminki, K. (2000). Environmental and heritable factors in the causation of cancer—analyses of cohorts of twins from Sweden, Denmark, and Finland. N. Engl. J. Med. *343*, 78–85.
37. Grady, W.M. (2003). Genetic testing for high-risk colon cancer patients. Gastroenterology *124*, 1574–1594.
38. Tiernan, J.P., Perry, S.L., Verghese, E.T., West, N.P., Yeluri, S., Jayne, D.G., and Hughes, T.A. (2013). Carcinoembryonic antigen is the preferred biomarker for in vivo colorectal cancer targeting. Br. J. Cancer *108*, 662–667.
39. Kim, T.M., Laird, P.W., and Park, P.J. (2013). The landscape of microsatellite instability in colorectal and endometrial cancer genomes. Cell *155*, 858–868.

40. Hensel, J.A., Chanda, D., Kumar, S., Sawant, A., Grizzle, W.E., Siegal, G.P., and Ponnazhagan, S. (2011). LL-37 as a therapeutic target for late stage prostate cancer. Prostate *71*, 659–670.

41. Lee, J.H., Isayeva, T., Larson, M.R., Sawant, A., Cha, H.R., Chanda, D., Chesnokov, I.N., and Ponnazhagan, S. (2015). Endostatin: a novel inhibitor of androgen receptor function in prostate cancer. Proc. Natl. Acad. Sci. USA *112*, 1392–1397.

42. Hensel, J.A., Khattar, V., Ashton, R., and Ponnazhagan, S. (2018). Characterization of immune cell subtypes in three commonly used mouse strains reveals gender and strain-specific variations. Lab. Invest. *99*, 93–106.

43. Cha, H.R., Lee, J.H., Hensel, J.A., Sawant, A.B., Davis, B.H., Lee, C.M., Deshane, J.S., and Ponnazhagan, S. (2016). Prostate cancer-derived cathelicidin-related antimicrobial peptide facilitates macrophage differentiation and polarization of immature myeloid progenitors to protumorigenic macrophages. Prostate *76*, 624–636.

OMTO, Volume *12*

Supplemental Information

Recombinant AAV-CEA Tumor Vaccine in Combination with an Immune Adjuvant Breaks Tolerance and Provides Protective Immunity

Jonathan A. Hensel, Vinayak Khattar, Reading Ashton, and Selvarangan Ponnazhagan

Supplemental Figure 1

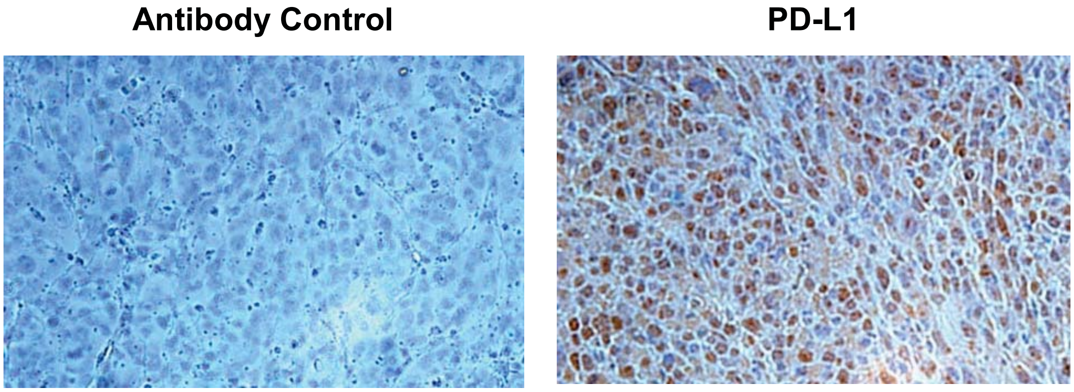

PD-L1 expression in MC38-CEA tumor. Representative immunohistochemistry analysis indicates high expression of PD-L1 in a mouse with resistant tumor growth, within the rAAV-CEA treatment group.
